# Water desalination with a single-layer MoS_2_ nanopore

**DOI:** 10.1038/ncomms9616

**Published:** 2015-10-14

**Authors:** Mohammad Heiranian, Amir Barati Farimani, Narayana R. Aluru

**Affiliations:** 1 Department of Mechanical Science and Engineering, Beckman Institute for Advanced Science and Technology, University of Illinois at Urbana-Champaign, Urbana, Illinois 61801, USA

## Abstract

Efficient desalination of water continues to be a problem facing the society. Advances in nanotechnology have led to the development of a variety of nanoporous membranes for water purification. Here we show, by performing molecular dynamics simulations, that a nanopore in a single-layer molybdenum disulfide can effectively reject ions and allow transport of water at a high rate. More than 88% of ions are rejected by membranes having pore areas ranging from 20 to 60 Å^2^. Water flux is found to be two to five orders of magnitude greater than that of other known nanoporous membranes. Pore chemistry is shown to play a significant role in modulating the water flux. Pores with only molybdenum atoms on their edges lead to higher fluxes, which are ∼70% greater than that of graphene nanopores. These observations are explained by permeation coefficients, energy barriers, water density and velocity distributions in the pores.

## Abstract (web summary)

Nanopores in two-dimensional materials are arousing considerable interest as filtration membranes to solve the problem of providing fresh water to a growing population. Here, the authors evaluate the potential of single-layer MoS_2_ to effectively reject salt ions whilst maintaining high flow rates.

## Graphical abstract

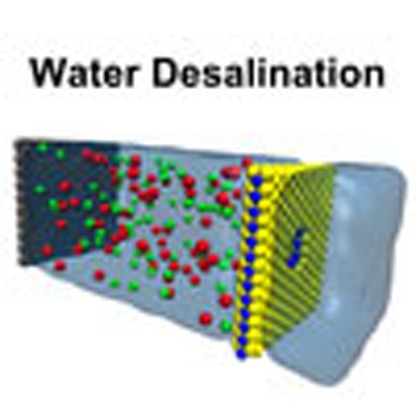

Producing fresh water is currently a great challenge facing the society^1^,^2^,^3^,^4^. High capital costs and low efficiency of current desalination technology motivate the need for advances in desalination technology^5^,^6^. Approximately, half of the current desalination plants use reverse osmosis (RO) technologies^2^,^5^. RO based on traditional polymeric membranes faces several challenges including slow water transport^7^,^8^. Advances in nanotechnology open up opportunities to design energy-efficient membranes for water desalination^9^,^10^. Nanopores with diameters ranging from a few Angstroms to several nanometres can be drilled in membranes to fabricate molecular sieves^11^,^12^,^13^. As the diameter of the nanopore approaches the size of the hydrated ions, various types of ions can be rejected by nanoporous membranes promising efficient water desalination. Among nanoscale materials, graphene and carbon nanotubes were extensively studied for both water transport and desalination^14^,^15^,^16^,^17^,^18^. Graphene, a single-atom-thick membrane (0.34 nm) was demonstrated to have several orders of magnitude higher flux rates compared with conventional zeolite membranes^6^,^11^,^15^,^16^,^19^,^20^. Since water flux through a membrane scales inversely with the membrane's thickness^11^, graphene is attractive over most other materials due to its single-atom thickness^12^,^16^.

It has been shown that chemical functionalization of a graphene nanopore (for example, adding hydroxyl groups) can enhance its permeability^19^,^20^, but reduces desalination efficiency^19^. Hydroxyl groups provide hydrophilic sites at the edge of the pore, which give rise to the attraction of water molecules and enhanced flux due to denser packing of water inside the pore^19^. Adding precise functional groups to the edge of nanopores requires complex fabrication^21^; therefore, identifying a single-atom-thick membrane with hydrophilic sites can lead to further advances in water desalination technology.

Recently, a nanopore in a single-layer molybdenum disulfide (MoS_2_) has been investigated for DNA sequencing and has been shown to provide better results compared with graphene nanopores^9^,^22^. Compared with graphene, a MoS_2_ single layer has two types of atoms, that is, molybdenum (Mo) and sulfur (S). A single-layer MoS_2_ has a thickness of ∼1.0 nm (ref. 23) and is a mechanically strong material with an effective Young's modulus of 270±100 GPa, that is comparable to that of steel^24^. The possibility to craft the pore edge with Mo, S or both provides flexibility to design the nanopore with desired functionality. Recently, it has been shown that a nozzle-like structure of protein channels and other nanoscale membranes enhances water permeation^25^. The fish-bone structure of MoS_2_ makes it amenable for a nozzle-like sub-nanometer pore for fast water permeation^25^.

Although theoretical studies of membrane efficiency are important in desalination technology, there are other aspects concerning fabrication and manufacturability of membranes such as large-area synthesis with defect-free, well-defined sealed membranes and precise pore generation that need to be addressed. Using a highly focused electron beam, and transmission electron microscope, versatile nanopores with diameters ranging from 1 to 10 nm were sculpted successfully in MoS_2_ membranes^9^. Waduge *et al*.^26^ reported that a large-area, well-sealed membrane with nanopores as tiny as 2.8 nm can be fabricated. Compared with graphene, the contamination of these membranes can be lower as carbon atoms in graphene are more susceptible to contamination during chemical vapour deposition (CVD) growth. Feng *et al*.^27^ also achieved high-quality scalable fabrication of nanopores in a single-layer MoS_2_ with sub-nanometre precision using electrochemical reaction. Several other studies have been performed on the synthesis of large-area MoS_2_ monolayers^28^,^29^,^30^,^31^,^32^,^33^,^34^,^35^,^36^,^37^. Recently, a few groups^29^,^34^,^37^ have successfully used CVD to produce highly crystalline MoS_2_ of centimetre dimensions. In another study^36^, a refined CVD method was proposed to create high-quality monolayer MoS_2_ crystals in which the grain boundaries of MoS_2_ were faceted more strongly than that of graphene resulting in mechanically more stable MoS_2_ monolayers. Membrane sealing also plays an essential role in the synthesis of large-area membranes required in desalination. Waduge *et al*.^26^ showed that their CVD approach resulted in almost fully sealed MoS_2_ membranes. Combination of these results^9^,^13^,^26^,^27^,^28^,^29^,^30^,^31^,^32^,^33^,^34^,^35^,^36^,^37^ and the recent focus on a single-layer MoS_2_ fabrication is promising for the large-scale manufacturing of a single-layer MoS_2_.

Here we demonstrate that a single-layer MoS_2_ can effectively separate ions from water. Using molecular dynamics simulations, we investigate water desalination in MoS_2_ as a function of pore size, chemistry, geometry and applied hydrostatic pressure.

## Results

### Water fluxes

**Figure 1:**
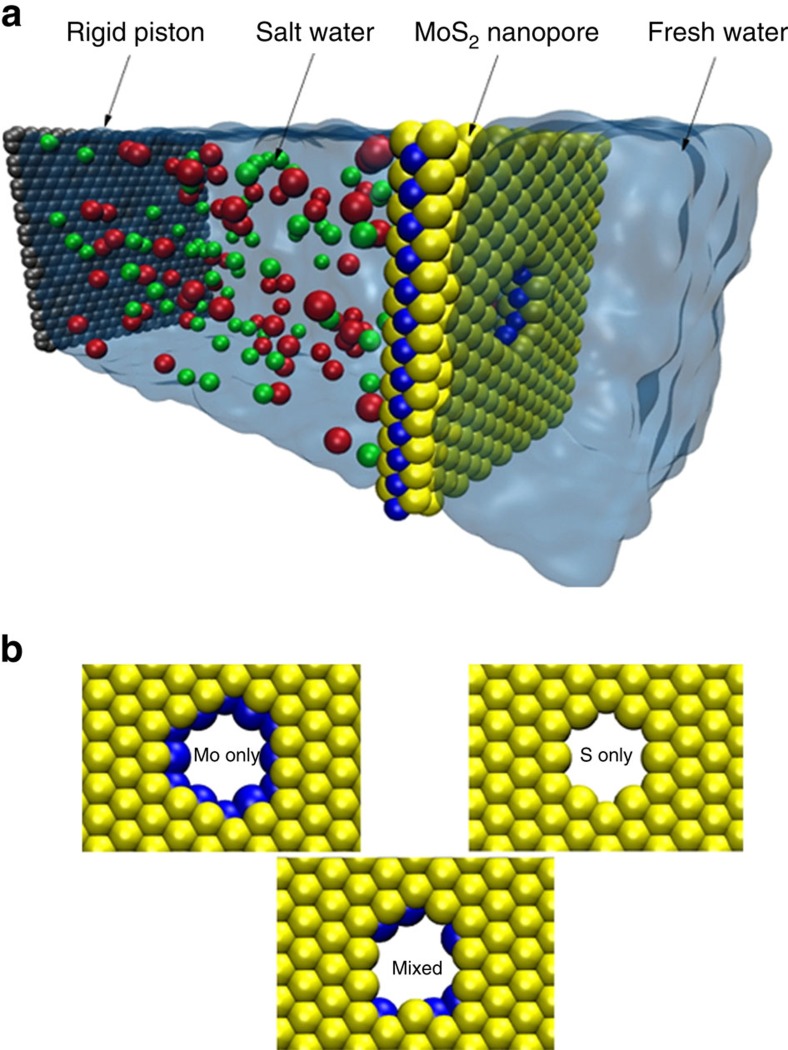
Simulation box and different pore architectures. (**a**) Schematic of the simulation box consisting of a MoS_2_ sheet (molybdenum in blue and sulfur in yellow), water (transparent blue), ions (in red and green) and a graphene sheet (in gray). (**b**) Left: Mo only pore type. Right: S only pore type. Bottom: mixed pore type.

**Figure 2:**
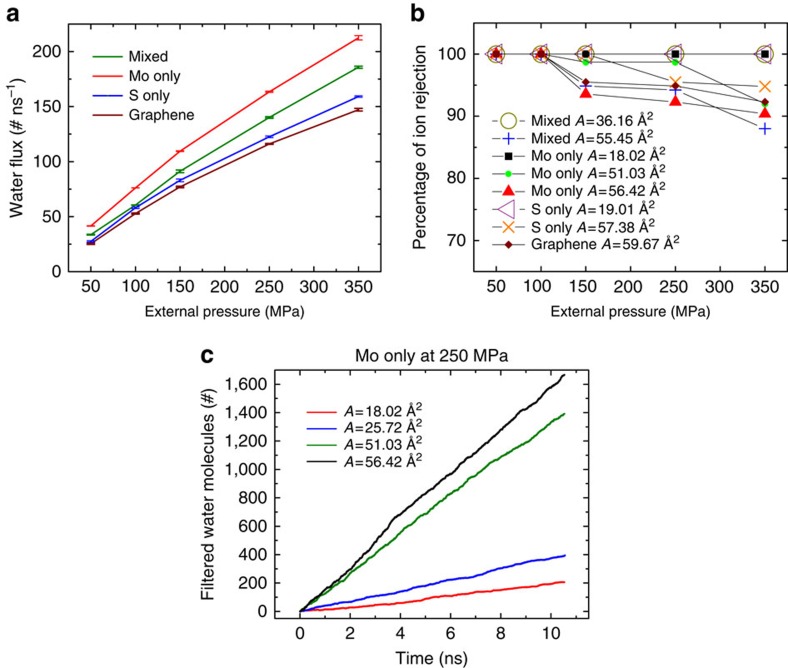
Water permeation and salt rejection. (**a**) Water flux as a function of the applied pressure for mixed, Mo only, S only and graphene nanopores with similar pore areas. (**b**) Percentage of ion rejection by various pores as a function of the applied pressure. Pores with different edge chemistries as well as various pore areas (denoted by *A*) are considered. (**c**) Number of water molecules (#) filtered through Mo only pores as a function of simulation time for different pore areas at a fixed pressure of 250 MPa.

A typical simulation box consists of a single-layer MoS_2_, a graphene sheet (acting as a rigid piston to apply the external pressure), water and ions (Fig. 1a). Here three pore edge types for MoS_2_ are considered to study the effect of terminating atoms and pore chemistry on the rate of water permeation and ion rejection. The first type of pore, which is labelled as mixed in this study, is a combination of molybdenum and sulfur atoms. The other two pore types are labelled as Mo only and S only, as these are terminated by molybdenum and sulfur atoms, respectively (Fig. 1b). Water fluxes through various MoS_2_ nanopores as a function of the applied pressure gradient are presented in Fig. 2a. Three MoS_2_ pore types (mixed, Mo only and S only) were studied to explore their rejection rate and flux. To investigate the relative performance of MoS_2_ over other two-dimensional materials, a graphene nanopore, which has been shown to be promising for water desalination, is also considered^11^,^19^. For the sake of comparison, the three MoS_2_ pores and the graphene pore have approximately equivalent accessible pore areas (mixed, *A*=55.45 Å^2^; Mo only, *A*=56.42 Å^2^; S only, *A*=57.38 Å^2^; and graphene, *A*=59.67Å^2^). Our results indicate that the Mo only pore has the highest rate of water permeation followed by the mixed, S only and the graphene pore for all the applied pressures (Fig. 2a). Water flux through the mixed pore is intermediary between Mo only and S only nanopores. The higher water fluxes through MoS_2_ nanopores compared with graphene nanopores imply that for a desired water flux, a smaller applied pressure is needed with MoS_2_ nanopores. Later, in this paper, we will explain the physical chemistry and geometrical foundations of MoS_2_ pore that give rise to a higher flux.

### Salt rejection efficiency

The other important aspect in water desalination is the ability of the membrane to reject ions. The percentage of total ions rejected by the MoS_2_ and graphene pores is plotted as a function of the applied pressure in Fig. 2b. The rejection is calculated after 1,700 water molecules have filtered through the pores for all pressures. Pore sizes ranging from 20 to 60 Å^2^ are considered for the three types of MoS_2_ pores. The ion rejection decreases at higher pressures as high pressures induce higher forces on the ions giving rise to more ion translocation events. The ion rejection of small pores (for example, 18.02 Å^2^) is found to be 100% for all types of pores. For larger pore sizes, ions escape through the pore reducing the rejection efficiency. For the pores with equivalent areas (mixed, *A*=55.45 Å^2^; Mo only, *A*=56.42 Å^2^; S only, *A*=57.38 Å^2^; and graphene, *A*=59.67 Å^2^), the general trend for ion rejection is quite similar regardless of the type of the pore (Fig. 2b). In other words, ion rejection is mainly dependent on the pore area and the type of the pore plays a less important role, for example, for the four pores considered, the difference in rejection is <10% even at a high pressure of 350 MPa.

As shown in Fig. 2c, the water filtration rate increases sharply as the pore area increases from ∼20 to ∼50 Å^2^. The sharp change in the water flow rate is due to the formation of single-file chain of water in small pores (∼20 Å^2^). As shown in ref. 11, the water flow rate is considerably reduced because of the weak hydrogen bonding in single-file chains. For efficient water desalination, pore sizes should be chosen such that both the ion rejection and water filtration rate are optimized since very small pores lack high permeation rates and large pores (wider than 60 Å^2^) fail to effectively reject ions.

As observed by Cohen-Tangui *et al*.^19^ for graphene, the polarizability of water also has a little effect on ion rejection in MoS_2_ nanopores. To introduce the effect of polarization, the flexible simple point charge (SPC/F) model^38^ was used. The ion rejection percentages associated with the flexible water model are within 2% of those modelled with the SPC/E water.

### Permeation coefficient

To quantify the water permeability through various pores, we compute the permeability coefficient, *p*, across the pore. For dilute solutions^39^,


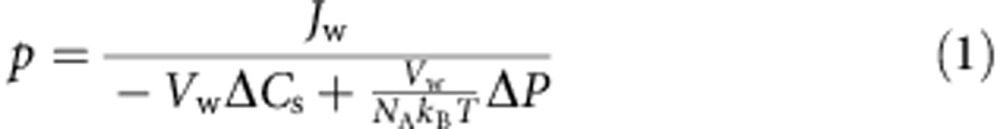


where *J*_w_ is the flux of water (# ns^−1^), *V*_w_ is the molar volume of water (18.91 ml mol^−1^), Δ*C*_s_ is the concentration gradient of the solute (1.0 M), *N*_A_ is the Avogadro number, *k*_B_ is the Boltzmann constant, *T* is the temperature (300 K) and Δ*P* is the applied hydrodynamic pressure (MPa). The permeability coefficients of the mixed, Mo only, S only and graphene pores were calculated to be 71.64, 83.61, 62.69 and 59.32 # ns^−1^, respectively. These coefficients are expected to also hold true for small applied pressures (<10 MPa), which are normally used in water desalination, since the relationship between the external pressure and the rate of water permeation is observed to be quite linear (Fig. 2a). Previous studies^40^,^41^ also show that water flux in small nanochannels is linear with respect to external pressure. The permeation rates through various pores (Mo only>mixed>S only>graphene) can also be explained by the energy barrier that a water molecule needs to overcome to enter the pore. These barriers were computed to be *ΔE*_Mo only_=8.50 *k*_B_*T*, *ΔE*_mixed_=8.84 *k*_B_*T*, *ΔE*_S only_=9.01 *k*_B_*T*, *ΔE*_graphene_=11.05 *k*_B_*T*, which are consistent with the results in Fig. 2a. The details on the energy barrier calculations are documented in Supplementary Fig. 1.

### Physical chemistry and geometry of the pore

Water flux (*Q*) is a function of density (*ρ*) inside the pore, velocity (*V*) of water through the pore and the area of the pore (*A*), (*Q*=*ρ*·*V*·*A*). In water desalination, increasing the area of the pore limits the salt rejection capability of the pore. As the area of the pore increases, the efficiency of rejection decreases^25^, leaving *ρ* and *V* as the control parameters to increase water flux through the pore.

**Figure 3:**
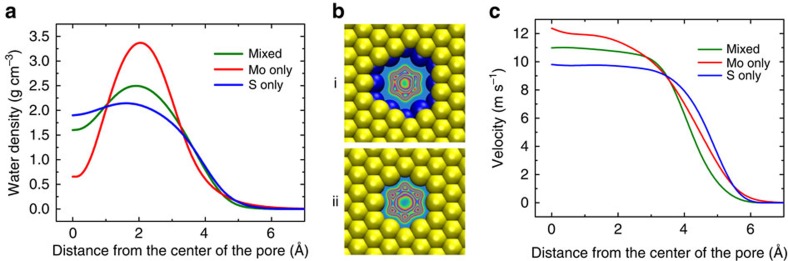
Water density and velocity profiles. (**a**) Water density distribution in the radial direction in the mixed, Mo only and S only pores with equivalent pore sizes (mixed, *A*=55.45 Å^2^ ; Mo only, *A*=56.42 Å^2^; S only, *A*=57.38 Å^2^) at a fixed pressure of 250 MPa. (**b**) Density map of water distribution in Mo only (i) and S only (ii) pores. Blue denotes a zero probability of finding a water molecule and red indicates the highest probability of observing a water molecule. (**c**) Axial velocity of water molecules in the radial direction for mixed, Mo only and S only nanopores.

As shown above, Mo only pore exhibits the highest rate of water permeation. This can be explained by the higher water density (*ρ*) and velocity (*V*) in the Mo only pore compared with those of the S only and mixed pores (Fig. 3a–c). The average density of water follows the order of Mo only>mixed>S only (1.47, 1.37 and 1.31 g cm^−3^, respectively). The denser packing of water molecules at the Mo only pore can be attributed to the hydrophilic nature of Mo sites^42^ at the edge of the nanopore, which attracts water molecules to the pore interior. It has been shown that the molybdenum surface has a water contact angle close to 0° (molybdenum is a transition metal with a large atomic diameter)^42^. Attraction of water molecules towards Mo sites becomes more obvious by comparing the mixed and S only pores densities (Fig. 3a). In the mixed pore, the existence of 50% Mo sites gives rise to higher density in the centre of the pore compared with that of S only pore (Fig. 3a).

Next, we explored the velocity profiles in the pore for all the three different pores. The velocities are also higher in Mo only pores compared with mixed and S only pores (Fig. 3c). The average velocity of water is 8.26, 7.53 and 7.51 m s^−1^ for Mo only, mixed and S only pores, respectively.

**Figure 4:**
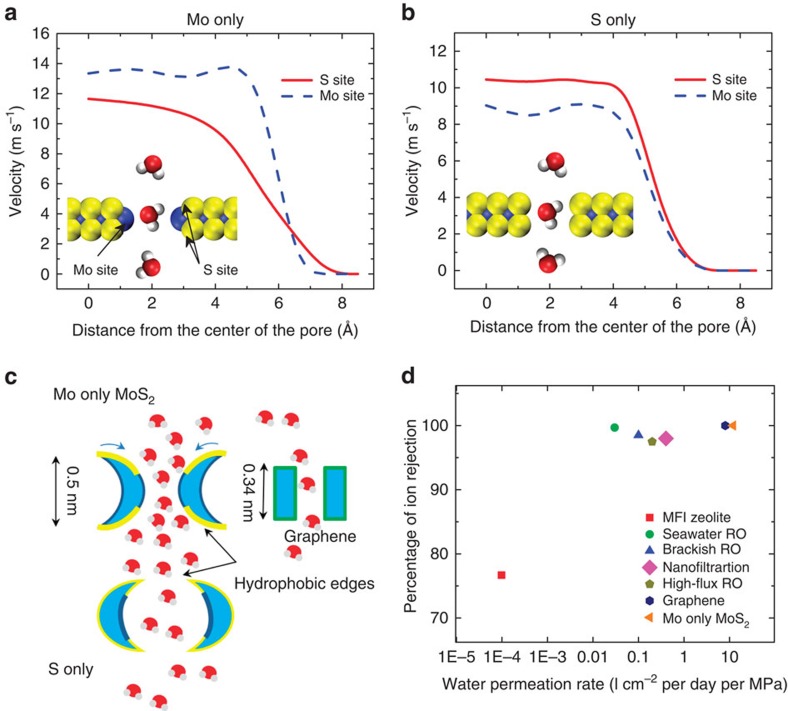
Effect of pore type on water permeation and salt rejection. (**a**) Axial velocity of water molecules in the radial direction at the location of S and Mo atom layers in the Mo only nanopore of *A*=56.42 Å^2^ at 250 MPa. (**b**) Axial velocity of water molecules in the radial direction at the location of S and Mo atom layers in the S only nanopore of *A*=57.38 Å^2^ at 250 MPa. (**c**) Cartoon representation of the pore architecture for Mo only, S only and graphene nanopore. (**d**) Performance of various membranes in terms of their ion rejection and water permeation rate. Water permeation rate is expressed per unit area of the membrane and per unit pressure as l cm^−2^ per day per MPa.

To shed deeper insight into the physical understanding of why the velocity of Mo only pore is higher compared with mixed and S only pores, we computed velocity profiles at the sites of S and Mo for both pore types of Mo only and S only (Fig. 4a,b). This is achieved by binning both pore types at Mo and S sites and averaging velocity at each point for a large number of sets of simulations. We observed that in the Mo only pore, the velocity is higher at Mo site compared with the S sites. Unlike Mo only pore, we did not observe the velocities to be higher in Mo site in the S only pore, (Fig. 4a,b) which implies that the arrangement of Mo and S sites matter for velocity profiles (see Supplementary Fig. 2 for more evidence on geometry dependency of the velocity in the pore).

It has been shown that conical nanopores have higher fluxes and permeation rates^25^,^43^,^44^. Many biological nanopores, including aquaporin^25^,^45^,^46^, have an hourglass shape, which facilitates rapid water permeation^47^. Solid-state nanopores have also been designed for conical/hourglass shape to enhance solute and DNA transport^48^,^49^. Here in Mo only pores, due to the fish-bone structure of MoS_2_ (ref. 9), the pore can be tailored^13^,^27^ to an hourglass shape at sub-nanometre length scale (see cartoon representation of comparison between Mo only, S only and graphene pores in Fig. 4c). Mo only pore has a contraction centre with hydrophobic S sites at the entrance and S only pore has an expanding centre (Fig. 4c). Graphene has a flat entrance and exit geometry with a single-atom-type exposure at the pore surface^50^. Water molecules slip on the hydrophobic edges of S and are attracted by the hydrophilic sites of Mo at the pore centre in Mo only case. This arrangement of hydrophobic and hydrophilic atoms along with the conical shape of the pore enhances the flux of water. Also, the water flux highly correlates with the energy barrier of each pore type. The computed potential of mean force for water molecules in each pore type is the reflection of pore chemistry and geometry. In Mo only pore, the potential of mean force is the lowest because of the conical/hourglass and the hydrophobic–hydrophilic arrangement of the pore atoms (Supplementary Fig. 1). The fundamental advantage of Mo only pore architecture over other pores is the interplay of geometry and chemistry to produce a higher flux of water.

## Discussion

Ion rejection and water flux are the two important factors defining the effectiveness and performance of a water desalination membrane. In Fig. 4d, ion rejection and water permeation rate are plotted for various nanomembrane materials^51^ (MFI-type zeolite^52^, commercial polymeric seawater RO^53^, brackish RO^53^, nanofiltration^53^ and high-flux RO^53^) including MoS_2_ and graphene investigated in this work. As shown in Fig. 4d, water permeation rate is theoretically enhanced by five orders of magnitude using MoS_2_ compared with conventional MFI-type zeolite. Also, there is a 70% improvement in the permeation rate of MoS_2_ compared with graphene. In the study by Cohen-Tanugi *et al*.^19^, the permeation rate for graphene is shown to be higher than the rate we observed for graphene. This is because, in our simulations, the porosity (the ratio of the pore area to the membrane area) is smaller, which decreases the permeation rate per unit area of the membrane. In this work, the comparison of MoS_2_ and graphene is performed by keeping all conditions identical in the simulations. Thus, MoS_2_ is potentially an efficient membrane for water desalination.

We have also investigated the potential performance of other transition metal dichalcogenide (MoSe_2_, MoTe_2_, WS_2_, WSe_2_ and so on) membranes. It was found that the transition metal atom plays a more important role than the chalcogen atom in desalination. More specifically, varying the Lennard-Jones (LJ) parameters of the chalcogen atom does not lead to a significant change in the ion rejection and water permeation (Supplementary Fig. 3).

In conclusion, we have shown that MoS_2_ membranes are promising for water purification and salt rejection. Mo only pores perform the best among all possible MoS_2_ pore architectures. MoS_2_ nanopores with water accessible pore areas ranging from 20 to 60 Å^2^ strongly reject ions allowing <12% of the ions (depending on pore areas) to pass through the porous membranes even at theoretically high pressures of 350 MPa. The water permeation rates associated with these MoS_2_ porous membranes are found to be two to five orders of magnitude greater than that of currently used membrane materials (MFI-type zeolite, commercial polymeric seawater RO, brackish RO, nanofiltration and high-flux RO) and 70% better than the graphene nanopore. The fish-bone, hourglass architecture of Mo only pore with special arrangement of hydrophobic edges and hydrophilic centre within 1-nm length enhances water permeation to a large extent compared with its other counterparts.

## Methods

Molecular dynamics (MD) simulations were performed using the LAMMPS package^54^. The graphene sheet, which acts as a rigid piston to exert external pressure on saline water, along with the MoS_2_ sheet, water molecules and ions were created by the Visual Molecular Dynamics^55^. The saline water box was placed between the graphene and MoS_2_ sheet and pure water was added on the other side of the MoS_2_ sheet as shown in Fig. 1. A nanopore was drilled in MoS_2_ by removing the desired atoms. The accessible pore areas considered range from 20 to 60 Å^2^ (Supplementary Fig. 4 for details on pore area calculations). The system dimensions are 4 × 4 × 13 nm in *x*, *y* and *z*, respectively. The box contains ∼16,000 atoms and the ions (sodium and chloride) have a molarity of ∼1.0, which is higher than the usual salinity of seawater (0.599 M) because of the computational cost associated with low-salinity solutions.

The extended simple point charge water model was used and the SHAKE algorithm was employed to maintain the rigidity of the water molecule. For non-bonded interactions, the mixing rule was used to obtain the LJ parameters except for carbon–water interactions, which were modelled by the force-field parameters given in ref. 50. The LJ parameters are tabulated in Supplementary Table 1. The LJ cutoff distance was 12 Å. The long-range electrostatic interactions were calculated by the Particle Particle Particle Mesh^56^. Periodic boundary conditions were applied in all the three directions.

For each simulation, first, the energy of the system was minimized for 10,000 steps. Next, the system was equilibrated in constant number of particles, pressure and temperature (NPT) ensemble for 1 ns at a pressure of 1 atm and a temperature of 300 K. Graphene and MoS_2_ atoms were held fixed in space during equilibration and the NPT simulations allow water to reach its equilibrium density (1 g cm^−3^). Then, an additional constant number of particles, volume and temperature (NVT) simulation was performed for 2 ns to further equilibrate the system. Temperature was maintained at 300 K using the Nosé-Hoover thermostat with a time constant of 0.1 ps (refs 57, 58). Finally, the production non-equilibrium simulations were carried out in NVT ensemble for 10 ns where different external pressures were applied on the rigid graphene sheet (no longer frozen in space) to characterize the water filtration through the MoS_2_ nanopores (Supplementary Movie 1). In the production runs, the MoS_2_ atoms were again held fixed in space to study solely the water transport and ion rejection properties of MoS_2_. To accelerate the MD simulations and gather enough statistics in the 10-ns simulations, high external pressures ranging from 50 to 350 MPa were considered in this work. Trajectories of atoms were collected every picosecond to obtain the results. For accurate velocity calculations, however, the trajectories were dumped every femtosecond and the data were averaged over 25 sets of simulations with different initial thermal velocity distributions.

## Additional information

**How to cite this article:** Heiranian, M. *et al*. Water desalination with a single-layer MoS_2_ nanopore. *Nat. Commun.* 6:8616 doi: 10.1038/ncomms9616 (2015).

## Supplementary Material

Supplementary Information Supplementary Figures 1-4, Supplementary Table 1 and Supplementary References.

Supplementary Movie 1 Water Desalination across an MoS2 pore
