# Supplementary material for: Water desalination with a single-layer MoS2 nanopore
Source: Nat Commun. 2015 Oct 14;6:8616. doi: 10.1038/ncomms9616 (PMC4634321; doi:10.1038/ncomms9616)
Supplement: Supplementary Information — Supplementary Figures 1-4, Supplementary Table 1 and Supplementary References. [file ncomms9616-s1.pdf]

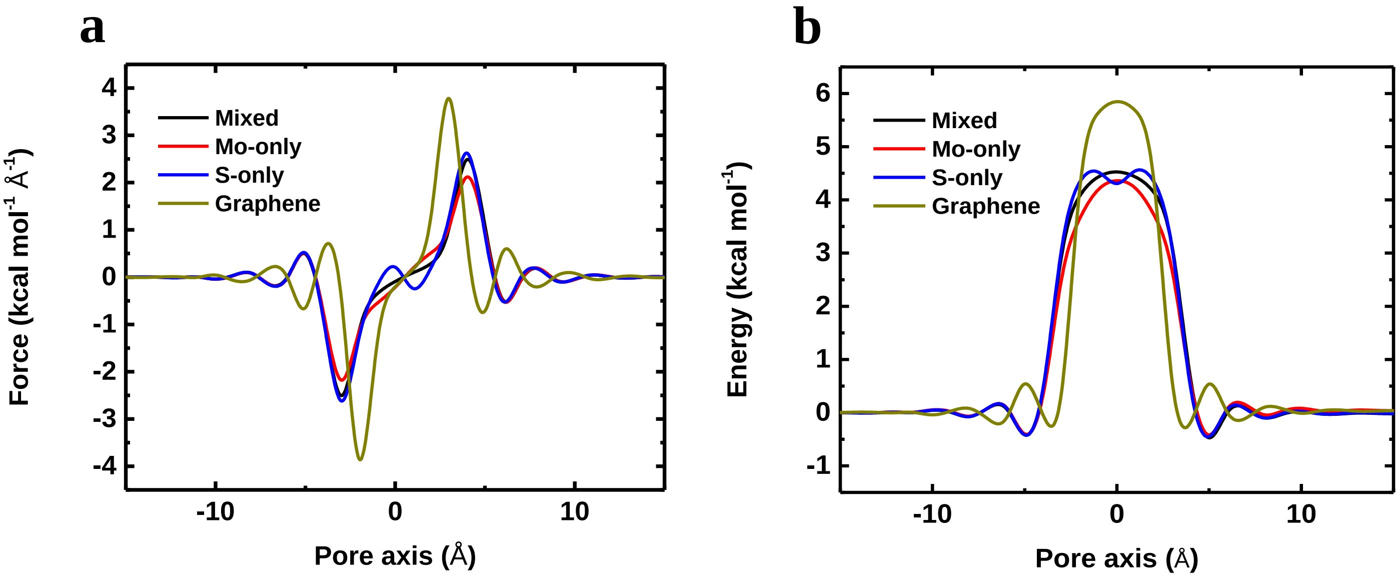

Supplementary Figure 1 | Energy Barriers. (**a**) Average force on a water molecule computed along the pore axis for the Mixed, Mo-only, S-only and graphene membranes with similar pore areas. (**b**) Potential of mean force computed along the pore axis for the Mixed, Mo-only, S-only and graphene membranes with similar pore areas. To calculate the energy barrier experienced by a water molecule when moving across a pore, the simulation box is first divided into bins of equal length along the axis of the pore (z). Next, in each bin, the force on each water molecule is averaged over both the simulation time and all the water molecules of the bin when the system is in equilibrium (no external pressure). Using the resulting average force (F) along the pore axis (z) (Supplementary Fig. 1a), the energy required to move a water molecule from a reference point (z_o) in the bulk water to any other point (z) can be obtained by $\int_{z_0}^{z} F(z)dz$.

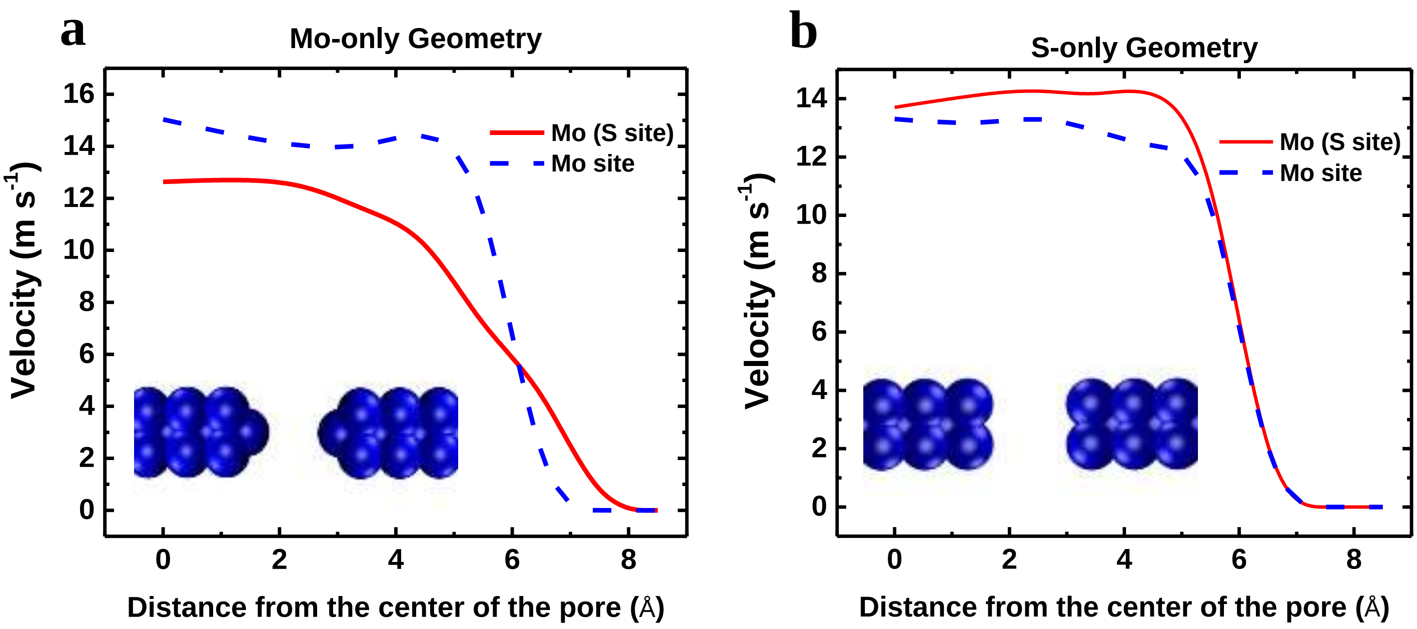

Supplementary Figure 2 | Velocities. Axial velocity profile of water molecules in the radial direction at the location of S and Mo atom layers where S atoms are replaced by Mo atoms (all Mo atoms). (**a**) For the Mo-only nanopore of Fig. 4a in the manuscript. (**b**) For the S-only nanopore of Fig. 4b in the manuscript. As discussed in the manuscript, the higher flux observed in the Mo-only pores is a result of the nozzle-like structure of the pore compared to the S-only pores. To further confirm the importance of the pore geometry in achieving the higher water velocities, the role of the atom type (Mo or S) is excluded by replacing all S atoms by Mo atoms (leading to a fictitious three-layer molybdenum membrane) as shown in Supplementary Fig. 2. The axial velocities of water in the radial direction at the location of each atom layer (the middle layer of Mo, and outer layers of Mo (S layers in real MoS_2)) are plotted in Supplementary Fig. 2a and Supplementary Fig. 2b for both Mo-only and S-only pore structures, respectively. Comparing these velocity profiles with those of the real MoS_2, in Fig. 4 of the manuscript, we notice that the general shape of the velocity profiles are identical meaning that the water flux is enhanced due to the nozzle-like geometry (hourglass shape) of the Mo-only pore and remains almost independent of the atom types.

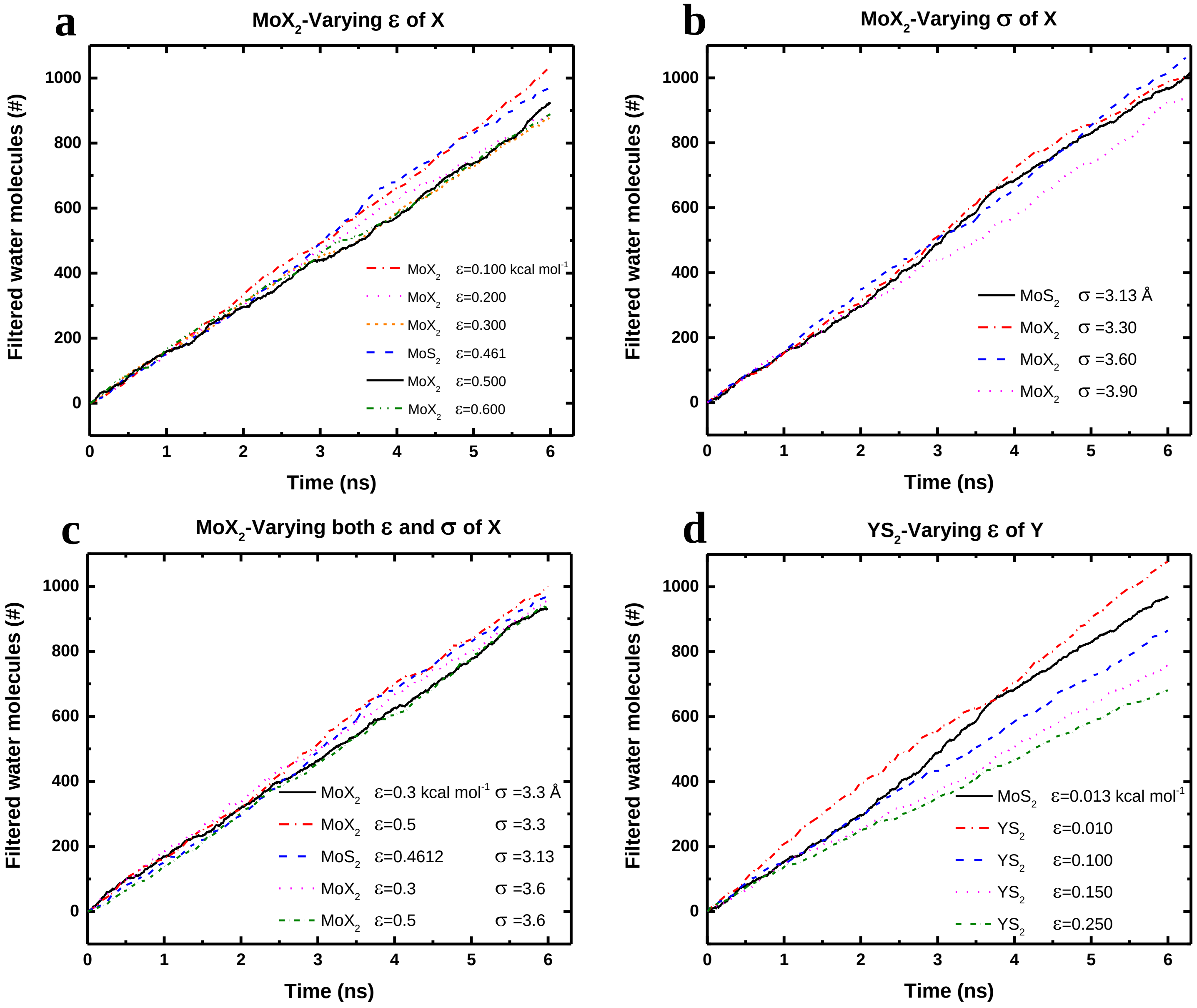

Supplementary Figure 3 | Other Transition Metal Dichalcogenide Membranes. Water permeation across a Mo-only geometry pore at 250 MPa for (**a**) MoX_2 by varying ε of X (**b**) MoX_2 by varying σ of X **c|** MoX_2 by varying both ε and σ of X (**d**) YS_2 by varying ε of Y. The molecular dynamics forcefield parameters are not available for the other transition metal dichalcogenide materials (TMD). Therefore, we swept over the Lennard-Jones parameters (σ, ε) of MoS_2 to investigate the potential performance of other TMD materials. Two different types of materials (MoX_2 and YS_2) were considered. For the MoX_2, only the parameters of the chalcogen atom (X) were varied to resemble the possible properties of membranes like $MoSe_2$ and $MoTe_2$. The Mo-only pore geometry was used and a pressure of 250 MPa was applied. As shown in Supplementary Fig. 3 (part a, b and c), the water permeation rate does not change significantly with varying σ and ε of X. Since atomic size of sulfur is smaller than those of the other chalcogen atoms

(Se, Te, etc.), only higher values of σ were considered. For the other type, YS_2, the ε of the transition metal (Y) atom was varied to study the efficiency of YS_2 membranes (Supplementary Fig. 3d). We did not change σ, since the pore area changes for Mo-only pore geometry. As shown in Supplementary Fig. 3d, changing the parameter of Y effects the permeation rate of water which decreases with increasing ε. The ion rejection percentages of MoX_2 and YS_2 do not change significantly and lie within 3% of ion rejection of MoS_2 (92%). Based on the analysis, we conclude that the transition metal atom plays a more important role than the chalcogen atom when it comes to choosing the best TMD material for desalination.

a

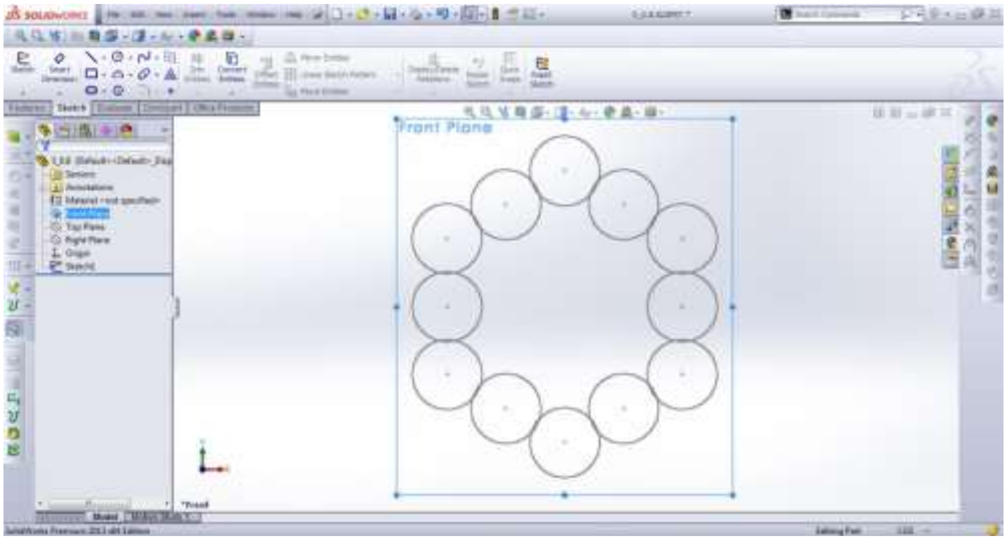

b

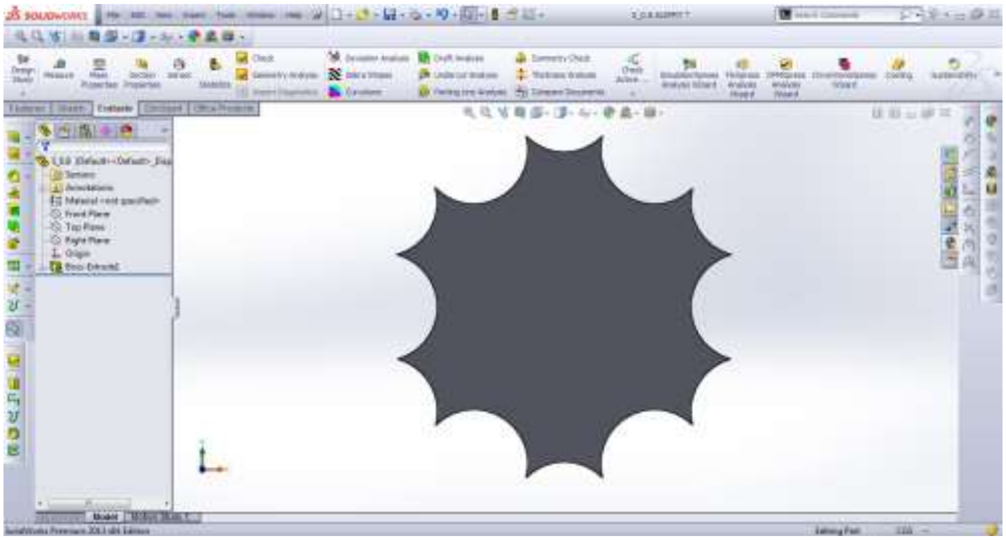

Supplementary Figure 4 | Calculation of pore area. (**a**) Terminating atoms of a pore represented by their size. (**b**) Extruded area of the pore. The pore area, which is accessible to water molecules, is computed by considering the size of the atoms on the edge of each pore. First, the coordinates of all terminating atoms' centers as well as the van der Waals radii of sulfur and molybdenum are input into the SolidWorks program as shown in Supplementary Fig. 4a. Then the accessible area is extruded through the atoms (Supplementary Fig. 4b) and the pore area is calculated.

Supplementary Table 1 | The Lennard-Jones parameters employed in the simulations are tabulated below.

Interaction	σ [Å]	ϵ [kcal mol^{-1}]
C-C[1]	3.3900	0.0692
Mo-Mo[2]	4.2000	0.0135
S-S[2]	3.1300	0.4612
O-O[1]	3.1656	0.1554
H-H[1]	0.0000	0.0000
Na-Na[3]	2.1600	0.3526
Cl-Cl[3]	4.8305	0.0128
C-O[4]	3.4360	0.0850
C-H[4]	2.6900	0.0383
Rest	Obtained by Lorentz-Berthelot rules.	

Supplementary References

1 Farimani, A. B. & Aluru, N. R. Spatial diffusion of water in carbon nanotubes: from fickian to ballistic motion. *J. Phys. Chem. B* **115**, 12145-12149 (2011).

2 Liang, T., Phillpot, S. R. & Sinnott, S. B. Parametrization of a reactive many-body potential for Mo-S systems. *Phys. Rev. B* **79**, 245110 (2009).

3 Joung, I. S. & Cheatham, T. E. Determination of alkali and halide monovalent ion parameters for use in explicitly solvated biomolecular simulations. *J. Phys. Chem. B* **112**, 9020-9041 (2008).

4 Wu, Y. B. & Aluru, N. R. Graphitic carbon-water nonbonded interaction parameters. *J. Phys. Chem. B* **117**, 8802-8813 (2013).
